# (1*R*,3*S*,5*R*,6*S*)-6-Acet­oxy-3-(4-methyl­phenyl­sulfon­yloxy)tropane

**DOI:** 10.1107/S1600536808035800

**Published:** 2008-11-13

**Authors:** Li-Min Yang, Liang Zhu, Yin-Yao Niu, Hong-Zhuan Chen, Yang Lu

**Affiliations:** a Department of Pharmacy, Shanghai Jiao Tong University School of Medicine, South Chongqing Road 280, Shanghai 200025, People’s Republic of China

## Abstract

In the title compound [systematic name: (1*R*,3*S*,5*R*,6*S*)-8-methyl-3-(4-methyl­phenyl­sulfon­yloxy)-8-aza­bicyclo­[3.2.1]octan-6-yl acetate], C_17_H_23_NO_5_S, the fused piperidine ring exists in a chair conformation with the N atom and one C atom displaced by 0.876 (2) and −0.460 (3) Å, respectively, on opposite sides of the mean plane defined by the other four atoms. The fused pyrrolidine ring adopts an envelope conformation with the N atom deviating by 0.644 (3) Å from the mean plane of the other four atoms.

## Related literature

For the synthesis, see: Yang & Wang (1998 ▶); Xie *et al.* (2005 ▶). For the pharmacological activity, see: Zhu *et al.* (2008 ▶).
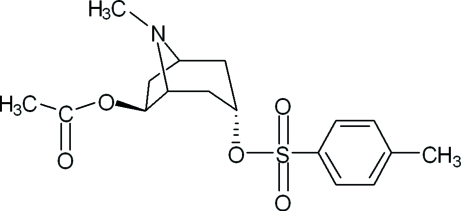


## Experimental

### 

#### Crystal data

C_17_H_23_NO_5_S
*M* *_r_* = 353.42
Orthorhombic, 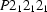

*a* = 6.9241 (6) Å
*b* = 15.5069 (14) Å
*c* = 16.1020 (15) Å
*V* = 1728.9 (3) Å^3^
*Z* = 4
Mo *K*α radiation
μ = 0.21 mm^−1^
*T* = 293 (2) K
0.47 × 0.41 × 0.31 mm

#### Data collection

Bruker SMART CCD area-detector diffractometer
Absorption correction: multi-scan (*SADABS*; Sheldrick, 2000 ▶) *T*_min_ = 0.751, *T*_max_ = 1.000 (expected range = 0.702–0.935)
10216 measured reflections
3770 independent reflections
3238 reflections with *I* > 2σ(*I*)
*R*_int_ = 0.066

#### Refinement

*R*[*F* ^2^ > 2σ(*F* ^2^)] = 0.040
*wR*(*F* ^2^) = 0.093
*S* = 0.97
3770 reflections
221 parameters
H-atom parameters constrained
Δρ_max_ = 0.20 e Å^−3^
Δρ_min_ = −0.23 e Å^−3^
Absolute structure: Flack (1983 ▶), 1671 Friedel pairs
Flack parameter: −0.01 (7)

### 

Data collection: *SMART* (Bruker, 2001 ▶); cell refinement: *SAINT* (Bruker, 2001 ▶); data reduction: *SAINT*; program(s) used to solve structure: *SHELXS97* (Sheldrick, 2008 ▶); program(s) used to refine structure: *SHELXL97* (Sheldrick, 2008 ▶); molecular graphics: *SHELXTL* (Sheldrick, 2008 ▶); software used to prepare material for publication: *SHELXTL*.

## Supplementary Material

Crystal structure: contains datablocks I, global. DOI: 10.1107/S1600536808035800/ng2508sup1.cif

Structure factors: contains datablocks I. DOI: 10.1107/S1600536808035800/ng2508Isup2.hkl

Additional supplementary materials:  crystallographic information; 3D view; checkCIF report

## Figures and Tables

**Fig. 1.**
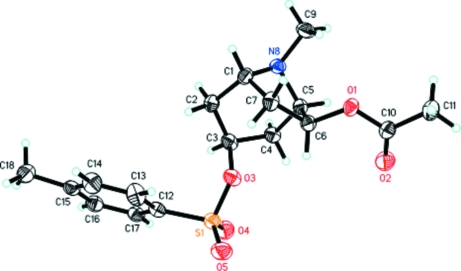
Ellipsoid plot.

## supplementary crystallographic information

### Comment

6β-Acetoxy-3α-paramethylbenzene sulfonyloxytropane, a racemic analog of Baogongteng A, prepared in our laboratory (Yang *et al.*, 1998), is a potent muscarinic receptor agonist and has been shown to be a promising candidate as a new antiglaucoma agent in our previous preclinical studies. Recently, we resolved the racemates and investigated the pharmacological characteristics of both enantiomers. The enantiomer (1*R*,3S,5*R*,6S) elicited agonistic activity on muscarinic receptors (Zhu *et al.*, 2008). We report here the crystal structure of the bioactive enantiomer. The three-dimensional structure of the title compound is shown in Fig.1. The absolute stereochemistry has been confirmed by the structure determination, with absolute structure parameter -0.01 (7) (Flack, 1983). The tropane ring system adopts a conformation typical of 3α-substituted derivatives, with the piperidine ring in a chair-like shape and the pyrrolidine ring in an envelope form with nitrogen atom as the flap. Atoms N and C3 are displaced by 0.8762(0.0024) and 0.4602(0.0031) Å on opposite sides of the plane containing four atoms C1,C2, C4 and C5 (plane I), and N is deviated by 0.6435 (0.0029)Å from the mean plane through the other four atoms C1,C5,C6,C7 (plane II). The phenyl group C12 to C17 is planar to within 0.0078 (plane III). The dihedral angles between planes I—II and planes I-III are 67.58 (0.09)° and 28.67 (0.08)° respectively.

### Experimental

Preparation of the title compound has been described previously (Yang *et al.*,1998). 6β-Acetoxy-3 α-tropanol (11 g, 0.06 mol) was dissolved in 20 ml CHCl3, and 4-toluene sulfonyl chloride (13 g, 0.07 mol) in 8 ml pyridine were added. The mixture was stirred at room temperature for 72 h. The solvent was evaporated in vacou. The residue was dissolved in anhydrous ethanol and recrystallized to give the hydrochloride of racemates of the title compound. Then it was dissolved in 20% ammonium hydroxide, extracted with dichloromethane and the organic phase was dried over anhydrous sodium sulfate and evaporated in vacou to give racemates of the title compound. The racemates (9.8 g, 0.03 mol) and (-)-2,3-dibenzoyl-*L*-tartaric acid (11 g, 0.03 mol) were dissolved in methanol for 2 h. After disposing at room temperature for 3 h, the (-)-2,3-dibenzoyl-*L*-tartarate as precipitate was collected by filtration and recrystallized from anhydrous ethanol. The salt was converted into the title compound as colorless crystals, 30% yield, m.p. 403–405 K, [α]_D_^20^ -11.42 (c = 0.1313, CHCl_3_), by treatment with 20% ammonium hydroxide as described above. The enantiomeric excess of the title compound was 98.05% (Xie *et al.*, 2005). Crystals suitable for X-ray analysis were obtained by slow crystallization from acetone.

### Refinement

The absolute configuration was assigned after refining the Flack parameter (Flack, 1983), using 1671 measured Friedel pairs. H atoms were placed in idealized positions, and refined as riding to their carrier atoms. with *U*_ĩso_(H) = 1.5*U*_eq_(methyl C) and *U*_iso_(H) = 1.2*U*_eq_(methylene and methine C).

### Crystal data

**Table:**

C_17_H_23_NO_5_S	*D*_x_ = 1.358 Mg m^−^^3^
*M**_r_* = 353.42	Melting point: 405 K
Orthorhombic, *P*2_1_2_1_2_1_	Mo *K*α radiation λ = 0.71073 Å
Hall symbol: P 2ac 2ab	Cell parameters from 3847 reflections
*a* = 6.9241 (6) Å	θ = 2.5–26.2º
*b* = 15.5069 (14) Å	µ = 0.21 mm^−^^1^
*c* = 16.1020 (15) Å	*T* = 293 (2) K
*V* = 1728.9 (3) Å^3^	P{rism, colorless
*Z* = 4	0.48 × 0.41 × 0.31 mm
*F*_000_ = 752	

### Data collection

**Table:**

Bruker SMART area-detector diffractometer	3770 independent reflections
Radiation source: fine-focus sealed tube	3238 reflections with *I* > 2σ(*I*)
Monochromator: graphite	*R*_int_ = 0.066
*T* = 293(2) K	θ_max_ = 27.0º
φ and ω scans	θ_min_ = 1.8º
Absorption correction: Multi-scan(SADABS; Sheldrick, 2000)	*h* = −6→8
*T*_min_ = 0.751, *T*_max_ = 1.000	*k* = −19→18
10216 measured reflections	*l* = −20→14

### Refinement

**Table:**

Refinement on *F*^2^	H-atom parameters constrained
Least-squares matrix: full	*w* = 1/[σ^2^(*F*_o_^2^) + (0.0451*P*)^2^] where *P* = (*F*_o_^2^ + 2*F*_c_^2^)/3
*R*[*F*^2^ > 2σ(*F*^2^)] = 0.040	(Δ/σ)_max_ = 0.001
*wR*(*F*^2^) = 0.093	Δρ_max_ = 0.20 e Å^−^^3^
*S* = 0.97	Δρ_min_ = −0.23 e Å^−^^3^
3770 reflections	Extinction correction: SHELXL97 (Sheldrick, 2008), Fc^*^=kFc[1+0.001xFc^2^λ^3^/sin(2θ)]^-1/4^
221 parameters	Extinction coefficient: 0.0065 (11)
Primary atom site location: structure-invariant direct methods	Absolute structure: Flack (1983), 1671 Friedel pairs
Secondary atom site location: difference Fourier map	Flack parameter: −0.01 (7)
Hydrogen site location: inferred from neighbouring sites	

### Special details

**Table:**

Geometry. All e.s.d.'s (except the e.s.d. in the dihedral angle between two l.s. planes) are estimated using the full covariance matrix. The cell e.s.d.'s are taken into account individually in the estimation of e.s.d.'s in distances, angles and torsion angles; correlations between e.s.d.'s in cell parameters are only used when they are defined by crystal symmetry. An approximate (isotropic) treatment of cell e.s.d.'s is used for estimating e.s.d.'s involving l.s. planes.
Refinement. Refinement of *F*^2^ against ALL reflections. The weighted *R*-factor *wR* and goodness of fit *S* are based on *F*^2^, conventional *R*-factors *R* are based on *F*, with *F* set to zero for negative *F*^2^. The threshold expression of *F*^2^ > σ(*F*^2^) is used only for calculating *R*-factors(gt) *etc*. and is not relevant to the choice of reflections for refinement. *R*-factors based on *F*^2^ are statistically about twice as large as those based on *F*, and *R*- factors based on ALL data will be even larger.

### Fractional atomic coordinates and isotropic or equivalent isotropic displacement parameters (Å^2^)

**Table:**

	*x*	*y*	*z*	*U*_iso_*/*U*_eq_	
S1	0.96500 (8)	0.56721 (3)	0.57026 (3)	0.04821 (16)	
O1	0.83603 (18)	0.75864 (9)	0.86913 (8)	0.0433 (3)	
O2	1.1550 (2)	0.76965 (12)	0.85445 (11)	0.0663 (5)	
O3	0.8477 (2)	0.62594 (8)	0.63122 (8)	0.0476 (4)	
O4	1.1038 (2)	0.61718 (11)	0.52631 (10)	0.0613 (4)	
O5	1.0274 (3)	0.49732 (10)	0.62094 (11)	0.0692 (5)	
N8	0.6078 (2)	0.83046 (10)	0.71495 (11)	0.0423 (4)	
C1	0.5182 (3)	0.74438 (13)	0.70783 (12)	0.0444 (5)	
H1	0.3790	0.7481	0.7183	0.053*	
C2	0.5559 (3)	0.71277 (14)	0.61955 (13)	0.0482 (5)	
H2A	0.5003	0.6558	0.6130	0.058*	
H2B	0.4918	0.7511	0.5807	0.058*	
C3	0.7683 (3)	0.70892 (12)	0.59874 (13)	0.0447 (5)	
H3	0.7838	0.7103	0.5383	0.054*	
C4	0.8843 (3)	0.78262 (13)	0.63697 (12)	0.0429 (5)	
H4A	1.0190	0.7657	0.6405	0.051*	
H4B	0.8762	0.8326	0.6010	0.051*	
C5	0.8130 (3)	0.80705 (12)	0.72301 (12)	0.0380 (4)	
H5	0.8878	0.8554	0.7453	0.046*	
C6	0.8169 (3)	0.72949 (12)	0.78367 (12)	0.0387 (4)	
H6	0.9185	0.6882	0.7689	0.046*	
C7	0.6163 (3)	0.68959 (13)	0.77502 (14)	0.0446 (5)	
H7A	0.5459	0.6928	0.8270	0.053*	
H7B	0.6250	0.6297	0.7579	0.053*	
C9	0.5348 (3)	0.87927 (14)	0.78624 (13)	0.0528 (5)	
H9A	0.5454	0.8448	0.8355	0.079*	
H9B	0.4019	0.8940	0.7771	0.079*	
H9C	0.6094	0.9310	0.7929	0.079*	
C10	1.0149 (3)	0.77628 (12)	0.89615 (13)	0.0434 (5)	
C11	1.0125 (3)	0.80429 (14)	0.98486 (14)	0.0530 (6)	
H11A	0.9534	0.7603	1.0182	0.079*	
H11B	0.9402	0.8568	0.9899	0.079*	
H11C	1.1425	0.8137	1.0035	0.079*	
C12	0.7863 (3)	0.53147 (12)	0.50065 (13)	0.0436 (5)	
C13	0.6276 (3)	0.48904 (15)	0.53244 (15)	0.0596 (6)	
H13	0.6197	0.4778	0.5891	0.072*	
C14	0.4820 (3)	0.46356 (14)	0.48051 (16)	0.0593 (6)	
H14	0.3746	0.4357	0.5025	0.071*	
C15	0.4917 (3)	0.47852 (12)	0.39596 (14)	0.0463 (5)	
C16	0.6542 (3)	0.51942 (13)	0.36492 (14)	0.0490 (5)	
H16	0.6642	0.5288	0.3080	0.059*	
C17	0.8019 (3)	0.54657 (12)	0.41651 (12)	0.0446 (5)	
H17	0.9096	0.5745	0.3949	0.054*	
C18	0.3260 (4)	0.45457 (15)	0.33942 (17)	0.0645 (7)	
H18A	0.2196	0.4930	0.3490	0.097*	
H18B	0.2863	0.3964	0.3508	0.097*	
H18C	0.3667	0.4590	0.2826	0.097*	

### Atomic displacement parameters (Å^2^)

**Table:**

	*U*^11^	*U*^22^	*U*^33^	*U*^12^	*U*^13^	*U*^23^
S1	0.0516 (3)	0.0486 (3)	0.0444 (3)	0.0070 (2)	−0.0010 (3)	−0.0041 (2)
O1	0.0368 (7)	0.0631 (8)	0.0300 (7)	−0.0006 (6)	−0.0012 (6)	0.0014 (6)
O2	0.0381 (8)	0.0992 (13)	0.0617 (10)	−0.0063 (9)	0.0096 (8)	−0.0149 (10)
O3	0.0639 (9)	0.0453 (7)	0.0337 (7)	0.0072 (7)	0.0015 (7)	0.0006 (6)
O4	0.0454 (9)	0.0769 (10)	0.0615 (10)	−0.0050 (8)	0.0070 (8)	−0.0094 (9)
O5	0.0832 (12)	0.0597 (9)	0.0645 (11)	0.0239 (9)	−0.0145 (10)	−0.0013 (8)
N8	0.0422 (9)	0.0490 (9)	0.0358 (9)	0.0084 (7)	0.0006 (8)	−0.0016 (8)
C1	0.0341 (10)	0.0591 (12)	0.0400 (11)	0.0006 (9)	−0.0015 (9)	−0.0016 (9)
C2	0.0490 (12)	0.0551 (12)	0.0406 (11)	0.0020 (10)	−0.0097 (10)	−0.0017 (10)
C3	0.0588 (13)	0.0453 (11)	0.0301 (9)	0.0059 (9)	0.0002 (9)	0.0026 (9)
C4	0.0467 (11)	0.0474 (11)	0.0346 (10)	0.0008 (9)	0.0078 (9)	0.0048 (9)
C5	0.0385 (11)	0.0415 (10)	0.0341 (10)	−0.0026 (8)	0.0017 (9)	−0.0003 (8)
C6	0.0398 (11)	0.0459 (10)	0.0304 (9)	0.0017 (9)	0.0017 (8)	0.0026 (9)
C7	0.0438 (11)	0.0538 (12)	0.0361 (10)	−0.0082 (9)	0.0008 (9)	0.0032 (9)
C9	0.0546 (13)	0.0579 (12)	0.0458 (12)	0.0143 (10)	0.0057 (11)	−0.0063 (10)
C10	0.0411 (12)	0.0453 (11)	0.0439 (11)	−0.0006 (9)	−0.0037 (9)	0.0036 (9)
C11	0.0530 (14)	0.0634 (13)	0.0426 (12)	−0.0007 (11)	−0.0101 (11)	0.0016 (10)
C12	0.0497 (12)	0.0388 (10)	0.0424 (11)	0.0039 (9)	0.0068 (10)	−0.0026 (9)
C13	0.0732 (16)	0.0603 (14)	0.0453 (13)	−0.0174 (12)	0.0081 (12)	0.0037 (11)
C14	0.0588 (15)	0.0557 (12)	0.0636 (15)	−0.0159 (11)	0.0146 (13)	0.0027 (11)
C15	0.0481 (13)	0.0327 (9)	0.0581 (12)	0.0053 (9)	0.0031 (10)	−0.0041 (9)
C16	0.0544 (13)	0.0507 (11)	0.0420 (11)	0.0044 (10)	0.0067 (10)	−0.0022 (10)
C17	0.0444 (11)	0.0463 (11)	0.0433 (12)	0.0013 (9)	0.0126 (9)	−0.0023 (9)
C18	0.0541 (14)	0.0594 (14)	0.0800 (18)	−0.0012 (11)	−0.0065 (13)	0.0006 (13)

### Geometric parameters (Å, °)

**Table:**

S1—O4	1.4231 (16)	C6—H6	0.9800
S1—O5	1.4239 (16)	C7—H7A	0.9700
S1—O3	1.5663 (14)	C7—H7B	0.9700
S1—C12	1.759 (2)	C9—H9A	0.9600
O1—C10	1.341 (2)	C9—H9B	0.9600
O1—C6	1.454 (2)	C9—H9C	0.9600
O2—C10	1.184 (2)	C10—C11	1.493 (3)
O3—C3	1.494 (2)	C11—H11A	0.9600
N8—C9	1.465 (2)	C11—H11B	0.9600
N8—C5	1.472 (2)	C11—H11C	0.9600
N8—C1	1.476 (3)	C12—C13	1.379 (3)
C1—C2	1.526 (3)	C12—C17	1.379 (3)
C1—C7	1.534 (3)	C13—C14	1.368 (3)
C1—H1	0.9800	C13—H13	0.9300
C2—C3	1.510 (3)	C14—C15	1.383 (3)
C2—H2A	0.9700	C14—H14	0.9300
C2—H2B	0.9700	C15—C16	1.385 (3)
C3—C4	1.527 (3)	C15—C18	1.511 (3)
C3—H3	0.9800	C16—C17	1.383 (3)
C4—C5	1.519 (3)	C16—H16	0.9300
C4—H4A	0.9700	C17—H17	0.9300
C4—H4B	0.9700	C18—H18A	0.9600
C5—C6	1.550 (3)	C18—H18B	0.9600
C5—H5	0.9800	C18—H18C	0.9600
C6—C7	1.526 (3)		
			
O4—S1—O5	119.62 (11)	C5—C6—H6	111.5
O4—S1—O3	110.21 (9)	C6—C7—C1	104.05 (16)
O5—S1—O3	103.94 (9)	C6—C7—H7A	110.9
O4—S1—C12	109.26 (10)	C1—C7—H7A	110.9
O5—S1—C12	109.81 (10)	C6—C7—H7B	110.9
O3—S1—C12	102.57 (9)	C1—C7—H7B	110.9
C10—O1—C6	117.04 (15)	H7A—C7—H7B	109.0
C3—O3—S1	118.18 (12)	N8—C9—H9A	109.5
C9—N8—C5	113.03 (17)	N8—C9—H9B	109.5
C9—N8—C1	112.53 (16)	H9A—C9—H9B	109.5
C5—N8—C1	100.92 (14)	N8—C9—H9C	109.5
N8—C1—C2	106.91 (17)	H9A—C9—H9C	109.5
N8—C1—C7	105.06 (16)	H9B—C9—H9C	109.5
C2—C1—C7	113.78 (17)	O2—C10—O1	123.71 (19)
N8—C1—H1	110.3	O2—C10—C11	125.2 (2)
C2—C1—H1	110.3	O1—C10—C11	111.09 (18)
C7—C1—H1	110.3	C10—C11—H11A	109.5
C3—C2—C1	112.71 (17)	C10—C11—H11B	109.5
C3—C2—H2A	109.1	H11A—C11—H11B	109.5
C1—C2—H2A	109.1	C10—C11—H11C	109.5
C3—C2—H2B	109.1	H11A—C11—H11C	109.5
C1—C2—H2B	109.1	H11B—C11—H11C	109.5
H2A—C2—H2B	107.8	C13—C12—C17	120.5 (2)
O3—C3—C2	108.34 (16)	C13—C12—S1	118.30 (17)
O3—C3—C4	108.07 (16)	C17—C12—S1	121.17 (16)
C2—C3—C4	113.17 (17)	C14—C13—C12	119.9 (2)
O3—C3—H3	109.1	C14—C13—H13	120.1
C2—C3—H3	109.1	C12—C13—H13	120.1
C4—C3—H3	109.1	C13—C14—C15	121.2 (2)
C5—C4—C3	112.55 (16)	C13—C14—H14	119.4
C5—C4—H4A	109.1	C15—C14—H14	119.4
C3—C4—H4A	109.1	C14—C15—C16	118.1 (2)
C5—C4—H4B	109.1	C14—C15—C18	121.0 (2)
C3—C4—H4B	109.1	C16—C15—C18	120.8 (2)
H4A—C4—H4B	107.8	C17—C16—C15	121.6 (2)
N8—C5—C4	107.15 (17)	C17—C16—H16	119.2
N8—C5—C6	105.28 (15)	C15—C16—H16	119.2
C4—C5—C6	112.08 (16)	C12—C17—C16	118.72 (19)
N8—C5—H5	110.7	C12—C17—H17	120.6
C4—C5—H5	110.7	C16—C17—H17	120.6
C6—C5—H5	110.7	C15—C18—H18A	109.5
O1—C6—C7	107.18 (16)	C15—C18—H18B	109.5
O1—C6—C5	110.90 (16)	H18A—C18—H18B	109.5
C7—C6—C5	103.96 (15)	C15—C18—H18C	109.5
O1—C6—H6	111.5	H18A—C18—H18C	109.5
C7—C6—H6	111.5	H18B—C18—H18C	109.5
			
O4—S1—O3—C3	47.59 (16)	N8—C5—C6—C7	24.8 (2)
O5—S1—O3—C3	176.95 (14)	C4—C5—C6—C7	−91.32 (19)
C12—S1—O3—C3	−68.65 (15)	O1—C6—C7—C1	120.02 (17)
C9—N8—C1—C2	162.61 (17)	C5—C6—C7—C1	2.53 (19)
C5—N8—C1—C2	−76.63 (18)	N8—C1—C7—C6	−29.1 (2)
C9—N8—C1—C7	−76.2 (2)	C2—C1—C7—C6	87.5 (2)
C5—N8—C1—C7	44.6 (2)	C6—O1—C10—O2	0.3 (3)
N8—C1—C2—C3	57.9 (2)	C6—O1—C10—C11	−179.30 (16)
C7—C1—C2—C3	−57.6 (2)	O4—S1—C12—C13	−173.64 (16)
S1—O3—C3—C2	128.33 (15)	O5—S1—C12—C13	53.3 (2)
S1—O3—C3—C4	−108.70 (15)	O3—S1—C12—C13	−56.72 (18)
C1—C2—C3—O3	82.9 (2)	O4—S1—C12—C17	5.3 (2)
C1—C2—C3—C4	−36.9 (2)	O5—S1—C12—C17	−127.77 (18)
O3—C3—C4—C5	−83.20 (19)	O3—S1—C12—C17	122.19 (17)
C2—C3—C4—C5	36.8 (2)	C17—C12—C13—C14	−1.7 (3)
C9—N8—C5—C4	−162.88 (16)	S1—C12—C13—C14	177.25 (17)
C1—N8—C5—C4	76.72 (17)	C12—C13—C14—C15	0.9 (4)
C9—N8—C5—C6	77.6 (2)	C13—C14—C15—C16	0.6 (3)
C1—N8—C5—C6	−42.76 (19)	C13—C14—C15—C18	−176.7 (2)
C3—C4—C5—N8	−57.7 (2)	C14—C15—C16—C17	−1.4 (3)
C3—C4—C5—C6	57.3 (2)	C18—C15—C16—C17	175.95 (18)
C10—O1—C6—C7	164.05 (16)	C13—C12—C17—C16	0.9 (3)
C10—O1—C6—C5	−83.1 (2)	S1—C12—C17—C16	−177.98 (15)
N8—C5—C6—O1	−90.05 (18)	C15—C16—C17—C12	0.6 (3)
C4—C5—C6—O1	153.80 (16)		
